# *Fasciola hepatica* is refractory to complement killing by preventing attachment of mannose binding lectin (MBL) and inhibiting MBL-associated serine proteases (MASPs) with serpins

**DOI:** 10.1371/journal.ppat.1010226

**Published:** 2022-01-10

**Authors:** Carolina De Marco Verissimo, Heather L. Jewhurst, József Dobó, Péter Gál, John P. Dalton, Krystyna Cwiklinski

**Affiliations:** 1 Centre for One Health and Ryan Institute, School of Natural Sciences, National University of Ireland Galway, Galway, Ireland; 2 Institute of Enzymology, Research Centre for Natural Sciences, Budapest, Hungary; Tufts University, Cummings School of Veterinary Medicine, UNITED STATES

## Abstract

The complement system is a first-line innate host immune defence against invading pathogens. It is activated via three pathways, termed Classical, Lectin and Alternative, which are mediated by antibodies, carbohydrate arrays or microbial liposaccharides, respectively. The three complement pathways converge in the formation of C3-convertase followed by the assembly of a lethal pore-like structure, the membrane attack complex (MAC), on the pathogen surface. We found that the infectious stage of the helminth parasite *Fasciola hepatica*, the newly excysted juvenile (NEJ), is resistant to the damaging effects of complement. Despite being coated with mannosylated proteins, the main initiator of the Lectin pathway, the mannose binding lectin (MBL), does not bind to the surface of live NEJ. In addition, we found that recombinantly expressed serine protease inhibitors secreted by NEJ (rFhSrp1 and rFhSrp2) selectively prevent activation of the complement via the Lectin pathway. Our experiments demonstrate that rFhSrp1 and rFhSrp2 inhibit native and recombinant MBL-associated serine proteases (MASPs), impairing the primary step that mediates C3b and C4b deposition on the NEJ surface. Indeed, immunofluorescence studies show that MBL, C3b, C4b or MAC are not deposited on the surface of NEJ incubated in normal human serum. Taken together, our findings uncover new means by which a helminth parasite prevents the activation of the Lectin complement pathway to become refractory to killing *via* this host response, in spite of presenting an assortment of glycans on their surface.

## Author summary

The mammalian complement system plays a central role in the defence against invasive pathogens. This response is initiated by recognition of specific molecules attached on the surface of bacteria, virus and parasites. Antigen-antibody complex, sugars and lipopolysaccharides are recognized by initiators of the Classical, Lectin and Alternative complement pathway, respectively, which leads to activation of the complement response and subsequent inflammation, opsonisation, damage and elimination of the invasive organism. Therefore, during infection with helminth parasites such as *Fasciola hepatica*, which is covered in antigenic sugars, it would be expected that complement attack, especially via the Lectin pathway, would eliminate them, thereby preventing establishment of infection. We discovered that the infective stage of *F*. *hepatica*, the newly excysted juvenile (NEJ), is resistant to the damaging effects of complement by using unique means that consists of: (1) preventing binding of the main initiator of this cascade, the mannose binding lectin (MBL), to the parasite surface; and (2) secreting two protease inhibitors (serpins), FhSrp1 and FhSrp2, that inactivate the main serine proteases involved in the Lectin pathway activation, namely MASPs. The elucidation of how *F*. *hepatica* and other helminths evade complement attack is a tractable approach for the discovery of novel anti-parasite interventions.

## Introduction

The complement system is the frontline immune defence against invading microorganisms and parasites [1, 2]. The three pathways that activate the complement system, namely the Classical, Lectin and Alternative, consist of more than 35 plasma and membrane-associated proteins organized in a well-balanced network. Many of these proteins are proenzymes (serine proteases) that, in turn, are activated or serve as substrates for a series of extracellular proteolytic cascades [3, 4]. While each of the three complement pathways is initiated in its own very specific way (antigen-antibody for Classical, glycans for Lectin, and bacterial lipopolysaccharides (LPS) for Alternative pathway), they all converge in the formation of C3-convertase (C4b2a in the Classical and Lectin pathways; C3bBb in the Alternative pathway) and result in the assembly of a pore-like structure, the membrane attack complex (MAC), that is inserted into the membrane of the target cell or pathogen and prompts their lysis [5]. Activation of complement attack via any of the three pathways also stimulates a strong pro-inflammatory response driven by anaphylatoxins (C3a, C4a and C5a) and opsonization of pathogens via binding of C3b and C4b fragments that are recognized by complement receptors on phagocytic cells [4, 5].

Remarkably, even though the complement system is present in tissues, fluids and blood, it often fails to kill protozoa and helminth parasites within these host compartments [1, 4], suggesting that parasites have developed effective mechanisms to evade or subvert this system. It is not surprising, therefore, that attention has been focused on uncovering parasite-specific mechanisms and molecules involved in this complement escape. Helminth parasites of the genus *Schistosoma* and *Echinococcus*, and protozoans of the genus *Trypanosoma* and *Leishmania* have been shown to avoid complement attack or complement mediated responses mainly by (1) avoiding recognition by complement activators, e.g., antibodies and mannose-binding lectins, (2) varying or changing their surface components, and (3) expressing regulators of complement activation as secreted or membrane-associated products [1, 4, 6–11].

Fasciolosis or liver fluke disease is a global neglected food- and water-borne infectious disease caused by the digenean trematode parasite *Fasciola hepatica*. It is widespread, affecting ~17 million people in more than 70 countries and a 50–75% of the domestic ruminants (sheep, cattle, water buffalo) depending on the region [12, 13], causing annual losses estimated at ~ €2.5 billion to livestock and food industry worldwide [14–16]. Mammalian hosts become infected by ingesting infective encysted metacercariae present on vegetation or floating in water. *F*. *hepatica* newly excysted juveniles (NEJ) emerge inside the host’s intestine and burrow through the gut wall into the peritoneal cavity to reach the liver, where they migrate through the parenchyma to find the bile ducts where they reside and reproduce for many years [17, 18].

The molecular mechanisms underlying the successful establishment and persistence of *F*. *hepatica* in the host involve sophisticated methods of modulating the host’s immune responses, including polarization towards a Th2 response, suppression of Th1/Th17 responses, alternative activation of macrophages (M2), induction of eosinophil apoptosis and inhibition of dendritic cells maturation [19–21]. What has yet to be investigated is whether NEJ possess a mechanism to prevent attack by the complement system as they enter the host. As a tissue-invasive pathogen, *F*. *hepatica* NEJ should be vulnerable to this rapid innate immune response. Furthermore, recent glycomic analysis has shown that the surface glycocalyx of both NEJ and adult fluke contains highly mannosylated glycans [22, 23], which would be expected to activate the complement system, specifically via the Lectin pathway. In this study, we aimed to understand how *F*. *hepatica* NEJ avoid the complement system during invasion of the mammalian host.

Here we show that *F*. *hepatica* NEJ are resistant to killing by complement. We discovered that NEJ selectively and potently inhibit the Lectin complement pathway. Our experiments point to an evasion strategy whereby *F*. *hepatica* NEJ, despite their mannosylated surface, prevent the binding of mannose binding lectin (MBL) on their surface. Concurrently, they express serine protease inhibitors, serpins, which regulate MBL-associated serine proteases (MASPs) that under normal circumstances play a role in generating the precursors of C3-convertase essential for the initiation of the Lectin pathway. This mechanism of complement avoidance has not been described before for any helminth and it may not be the only means by which *F*. *hepatica* blocks complement-mediated killing. Therefore, we place these mechanisms in the context of other putative tactics that the parasite may exploit to efficiently avoid complement attack.

## Materials and methods

### Ethical statement and samples

Samples of normal human sera (NHS) were obtained from healthy volunteers following ethical approval by the National University of Ireland Galway, Ireland, research ethics committee (R20.Jun.06). The samples were pooled and immediately stored at -80°C. All participants provided written informed consent prior to the study.

### Recombinant *F*. *hepatica* serpins (rFhSrp1 and rFhSrp2) and MASP proteins

The recombinant *F*. *hepatica* serpins, rFhSrp1 and rFhSrp2, were produced as described by De Marco Verissimo et al. [24]. Briefly, recombinant expression was carried out in *Escherichia coli* and recombinant proteins purified using Ni-NTA affinity chromatography (Qiagen). Protein concentration was measured by Bradford Protein Assay (Bio-Rad) and the proteins visualised on 4–20% SDS-PAGE gels (Bio-Rad) stained with BioSafe Coomassie (Bio-Rad). Polyclonal antibodies against rFhSrp1 and rFhSrp2 were produced in rabbits as previously described (Eurogentec; [24]). Recombinant MASP-1 catalytic region (CCP1-CCP2-SP; crMASP-1) and MASP-2 catalytic region (CCP1-CCP2-SP; crMASP-2) were produced as previous described [25, 26].

### Effect of live *F*. *hepatica* NEJ, ES, rFhSrp1 and rFhSrp2 on the Classical, Lectin and Alternative pathways of the complement system

*F*. *hepatica* newly excysted juveniles (NEJ) were obtained by excysting metacercariae (Italian and Aberystwyth isolates, Ridgeway Research Ltd), as previously described by Robinson et al. [27]. *F*. *hepatica* NEJ were cultured in RPMI-1640 media (ThermoFisher Scientific) supplemented with 30 mM HEPES (ThermoFisher Scientific), 0.1% glucose and 50 μg/ml gentamycin, at 37°C with 5% CO_2,_ for up to 24 hr. The culture media containing the excretory/secretory (ES) products was collected after 24 hr, concentrated using Amicon Ultra 3kDa columns (Merck Millipore) and stored at -80°C until use [28].

For the complement blocking assays, the NEJ were washed five times in Dulbecco’s phosphate-buffered saline (DPBS) (ThermoFisher Scientific) and cultured for further 1 hr in 100% NHS at 37°C, 5% CO_2_ (1 NEJ/1 μL). Samples of NHS alone were also incubated under the same conditions to be used as control sera (NHS-control). The cultured sera were recovered from each condition following the incubation and stored at -80°C until use in the complement assays.

Activation of the three complement pathways (Classical, Lectin and Alternative) was measured using the Wieslab Complement System Screen (Svar Life Science AB) in a 96-well plate format. Samples of NHS cultured with *F*. *hepatica* NEJ or NHS-control were diluted according the manufacturer’s instructions, in the required kit buffer, and incubated at room temperature (RT) for 15 min. Alternatively, to test the effect of the NEJ ES products, NHS was diluted in the required buffers and, after 15 min incubation, RPMI media or NEJ ES (20 μg) with or without the broad-spectrum cathepsin proteinase inhibitor E-64 (20 μM; Sigma-Aldrich) was added to the samples, which were incubated at RT for a further 25 min. The samples (100 μL) were then added to the wells of the Wieslab plates, incubated at 37°C for 1 hr and the activity of each complement pathway measured according to the manufacturer’s instructions. All the assays were performed in triplicate. The complement activity via each pathway, presented as a percentage, was calculated either relative to the activity within the NHS, NHS-control or within NHS assayed with RPMI only, set as 100% activity.

To test the effect of the recombinant *F*. *hepatica* rFhSrp1 and rFhSrp2 serpins on the complement pathways, NHS was diluted as above according to the manufacturer’s instructions, and incubated at RT for 15 min. Following which, rFhSrp1 (1 μM), rFhSrp2 (1 μM), rFhSrp1 and 2 combined (referred to hereon in as FhSrps, 1 μM), serine protease inhibitor Futhan (FUT-175; as a positive control in the recommended concentration 100 μM to inhibit complement pathways, BD-Pharmingen-Bioscience), or 1x PBS (negative control) was added to the NHS samples and incubated at RT for a further 25 min, before adding the samples to the wells of the Wieslab plates. The assays were developed as described above. All the assays were performed in triplicate and the percentage inhibition of each complement pathway was calculated relative to the activity within the control NHS samples with PBS.

### Immunolabeling of complement-derived proteins on the surface of *F*. *hepatica* NEJ

*F*. *hepatica* NEJ were excysted and cultured in RPMI-1640 media (ThermoFisher Scientific) for 23 hr, as described above. The media containing the NEJ was then supplemented with either 10% NHS, recombinant human MBL (rhMBL, 2 μg/mL; R&D Systems), or 1x PBS and incubated at 37°C, 5% CO_2_, for 1 hr. After five washes in DPBS, the NEJ were fixed in 4% paraformaldehyde (PFA) at RT for 4 hr for complement immunolocalization studies.

To analyse complement interaction and deposition on *F*. *hepatica* NEJ exposed to 10% NHS, the respective fixed parasites were washed three times in antibody diluent buffer (AbD) (0.1% Triton X-100 (*v/v*), 0.1% bovine serum albumin (*w/v*) and 0.1% sodium azide (*w/v*) in PBS) and probed overnight (ON) with rabbit anti-human MBL (1:250; Abcam), rabbit anti-human C3b (1:250; Aligent), rabbit anti-human C4b (1:250; Abcam), or rabbit anti-human C5b-9 (MAC) (1:500; Abcam), at 4°C. After three washes with AbD, fluorescein isothiocyanate (FITC)-labelled goat anti-rabbit IgG (1:200; Sigma-Aldrich) was added and the samples were incubated ON, in the dark at 4°C. Parasites incubated with 10% NHS were also probed directly with concanavalin A-FITC labelled (10 μg/mL; Sigma-Aldrich), ON at 4°C. The parasites were subsequently washed with AbD and counter-stained ON, in the dark at 4°C with phalloidin-tetramethylrhodamine isothiocyanate (TRITC) (200 μg/mL; Sigma-Aldrich).

In other experiments, live *F*. *hepatica* NEJ were cultured for 1 hr in the presence of recombinant human MBL (rhMBL, 2 μg/mL), after which the NEJ were washed, fixed and probed with rabbit anti-human MBL (1:250). Addition of the secondary FITC-antibody and counter-staining with TRITC were subsequently performed as described above.

*F*. *hepatica* NEJ cultured in RPMI alone and subsequently fixed, were washed with AbD and subsequently incubated with either rhMBL (2 μg/mL) or 10% NHS, for 1 hr at RT. After three washes with AbD, these samples were probed ON at 4°C with either rabbit anti-human MBL (1:250), rabbit anti-human C3b (1:250), rabbit anti-human C4b (1:250), or rabbit anti-human C5b-9 (MAC) (1:500). As non-related controls, samples of these NEJ fixed were probed with polyclonal rabbit anti-recombinant *F*. *hepatica* cathepsin L3 (rFhCL3, 1:500) followed by FITC-labelled goat anti-rabbit IgG secondary antibody (1:200; ThermoFisher Scientific). Alternatively, these NEJ parasites were probed directly with concanavalin A-FITC labelled (10 μg/mL), ON at 4°C. All these samples were counter-stained and processed as described above. The NEJ were mounted on slides in 10% glycerol (*v/v*) with 0.1M propyl gallate and covered with a coverslip. The slides were visualized in an Olympus Fluoview 3000 Laser Scanning Confocal Microscope under the PL APO CS 60x oil objective lens using Olympus type F immersion oil.

### Recognition of *F*. *hepatica* NEJ somatic extract by human mannose binding lectin

*F*. *hepatica* NEJ somatic extract was prepared by adding 100 μl RIPA buffer (Sigma-Aldrich) containing 5% protease and phosphatase inhibitor cocktail (Sigma-Aldrich) to ~1000 24 hr post-excystment NEJ. The sample was freeze-thawed three times and homogenised using a sterile pestle to extract the proteins. Following centrifugation at 10,000 x g for 40 min at 4°C, the supernatant was collected and the somatic protein concentration was measured by the Bradford Protein Assay (Bio-Rad).

#### hMBL-binding ELISA

Flat-bottom 96 well microtiter plates (Nunc MaxiSorp, Biolegend) were coated with mannan from *Saccharomyces cerevisiae* (2 μg/well; Sigma-Aldrich) or *F*. *hepatica* NEJ somatic extract (2 μg/well) in carbonate buffer ON, at 4°C. Recombinant human MBL (rhMBL, 2 μg/mL) or NHS (1:25) used as source of human MBL (hMBL), or PBS (as control) were diluted in dilution buffer (0.1% BSA (*w/v*), 1 M NaCl, 20 mM Tris, 10 mM CaCl_2_, pH 7.4) and added, in triplicate, to the mannan and NEJ somatic extract-coated wells and incubated ON, at 4°C. After three washes with wash buffer (120 mM NaCl, 10 mM Tris, 1 mM CaCl_2_, 0.05% Tween 20 (*v/v*), pH 7.4), the wells were treated with blocking buffer (0.1% BSA (*w/v*), 120 mM NaCl, 10 mM Tris, 1 mM CaCl_2_, pH 7.4) for 1 hr at 37°C. Bound hMBL was detected with rabbit anti-human MBL HRP-conjugated antibodies (1:4,000; US Biological Life Sciences). The assays were developed with 3.3’.5.5’ tetramethylbenzidine (TMB) substrate (Sigma-Aldrich) and the reaction was stopped by the addition of 100 μL 2N sulphuric acid to each well. Absorbance was measured at 450 nm in a PolarStar Omega Spectrophotometer (BMG LabTech, UK) and the OD450 intensity was considered proportional to the hMBL bound to the wells. To assess if *F*. *hepatica* serpins could interfere with the hMBL binding, rFhSrp1 (1 μM), rFhSrp2 (1 μM), rFhSrps (1 μM) or FUT-175 (100 μM) was added to the NHS samples prior to addition to the wells. These concentrations were conserved throughout all the experiments in this study, considering their effectiveness on inhibiting the lectin pathway assayed using the Wieslab test.

#### Detection of hMBL-binding molecules in *F*. *hepatica* NEJ somatic extract by transfer blotting

*F*. *hepatica* NEJ somatic extract (10 μg/lane) was resolved in a 4–20% SDS-PAGE gel and electro-transferred onto PVDF membrane. The membrane was incubated in blocking buffer (2% BSA (*w/v*) in PBST) and then probed ON at 4°C with either recombinant rhMBL (2 μg/mL) or NHS (1:25). After five washes, bound hMBL was detected with rabbit anti-human MBL HRP-conjugate antibodies (1:4,000) followed by developing with 3,3′-Diaminobenzidine substrate (DAB, Sigma-Aldrich). As controls, separate transfer strips containing the NEJ somatic extract (10 μg/lane) and rhMBL (1 μg/lane) were probed with the secondary antibody only.

### Assessing rFhSrp1 and rFhSrp2 interaction with serum-derived MASPs

We assessed the ability of the recombinant *F*. *hepatica* serpins (rFhSrp1 or rFhSrp2) to bind and block the activity of the native serum derived MASPs using two different assays.

#### ELISA assay to assess *F*. *hepatica* serpins interaction with serum-derived MASPs

The ELISA was performed according to Ferreira et al. [29] with slight modifications. Flat-bottom 96 well microtiter plates were coated with mannan from *S*. *cerevisiae* (5 μg/mL) in carbonate buffer (50 mM NaHCO_3_, 50 mM Na2CO_3_, pH 9.6). NHS diluted 1:100 in barbital buffer (4 mM barbital, 145 mM NaCl, 0.5 mM MgCl_2_, 2 mM CaCl_2_, 0.02% Tween 20 (*v/v*), 0.3% BSA (*w/v*), pH 7.4) was added to the mannan-coated plates and incubated for 2 hr at 37°C. After five washes with PBST containing 10 mM NaCl, either rFhSrp1 (1 μM), rFhSrp2 (1 μM), rFhSrps (1 μM) or 1x PBS was added to the wells and incubated at 37°C for 2 hr. Serpins bound to the wells were detected with rabbit anti-rFhSrp1 (1:1,000), anti-rFhSrp2 (1:1,000) or anti-rFhSrp1 and 2 mixed 1:1 (1:1,000), followed by a secondary antibody HRP-conjugated goat to rabbit anti-IgG (1:5,000). The assays were developed with TMB substrate and the reaction was stopped by the addition of 100 μL 2N sulphuric acid to each well. Absorbance was measured at 450 nm in a PolarStar Omega Spectrophotometer. All assays were carried out in triplicate and the OD450 intensity was considered proportional to the binding of the serpins.

#### Assessing the enzymatic activity of serum-derived MASPs

To investigate whether the *F*. *hepatica* recombinant serpins inhibit native serum-derived MASPs, samples of NHS diluted 1:10 in barbital buffer were incubated with either rFhSrp1 (1 μM), rFhSrp2 (1 μM), rFhSrps (1 μM), FUT-175 (100 μM) or PBS at RT for 25 min and then added to *S*. *cerevisiae* mannan-coated plates (20 μg/mL). The plates were incubated at 37°C for 5 min before the addition of the fluorogenic substrate, Z-Gly-Pro-Arg-AMC (20 μM; Bachem, UK). The proteolytic activity of serum derived MASPs was measured continuously over 1 hr at 37°C in a PolarStar Omega Spectrophotometer as relative fluorescent units (RFU). All assays were carried out in triplicate and the average activity, presented as a percentage, was calculated relative to the activity within the NHS-PBS sample, set as 100% activity. Serum MASPs activity was also determined in the NHS in which *F*. *hepatica* NEJ were cultured and compared to the NHS-control incubated without NEJ.

### Enzymatic activity of recombinant crMASP-1 and crMASP-2

In order to determine the optimal substrate to assess the enzymatic activity of recombinant crMASP-1 and crMASP-2, a panel of fluorogenic peptide substrates was screened against each enzyme (S1 Fig). The recombinant crMASP-1 (0.1 μM) or crMASP-2 (0.2 μM) was diluted in TBS-calcium buffer (150 mM NaCl, 50 mM Tris, 20 mM CaCl_2_, 0.05% Tween 20 (*v/v*), pH 7.8) before adding the fluorogenic substrate to initiate the reaction. The proteolytic activity was measured at 37°C continuously for up to 1 hr, as RFU in a PolarStar Omega Spectrophotometer. All assays were carried out in triplicate. From this data, the optimal substrates Z-Phe-Arg-AMC (40 μM; Bachem, UK) and Z-Ile-Glu-Gly-Arg-AMC (40 μM; Bachem, UK) were chosen to assess the activity of crMASP-1 and crMASP-2, respectively.

The inhibition assays were carried out using the same buffers and conditions as described above. The recombinant crMASP-1 or crMASP-2 was pre-incubated with 1 or 10 μM of either rFhSrp1, rFhSrp2 or FhSrps. The FUT-175 (100 μM) was used as positive control. All the samples were kept at 37°C for 15 min, before the substrate was added. The proteolytic activity of crMASPs was measured at 37°C continuously for up to 1 hr, as RFU in a PolarStar Omega Spectrophotometer. All the assays were performed in triplicate and the percentage inhibition of the crMASP-1 and crMASP-2 was calculated relative to the activity of each recombinant protein assayed alone.

### Quantification of C3 and C4 deposition by ELISA in the presence and absence of serpins

Mannose binding lectin (MBL)/MASP mediated complement activation was measured as described by Bultink et al. [30] with slight modifications. Briefly, flat-bottom 96 well microtitre plates were coated with mannan from *S*. *cerevisiae* (50 μg/mL) in carbonate buffer, washed three times with 1x PBS and incubated with blocking solution (0.5% BSA (*w/v*) in PBS) at RT for 2 hr. NHS diluted 1:100 in barbital buffer was incubated with either rFhSrp1 (1 μM), rFhSrp2 (1 μM), rFhSrps (1 μM), FUT-175 (100 μM) or 1x PBS, at RT for 20 min. NHS in which *F*. *hepatica* NEJ were cultured and NHS-control samples were also diluted and incubated in the same conditions. Subsequently, the samples were added, in triplicate, to the plates and incubated at 37°C for 1 hr. The C3 or C4 deposition onto the surface of the plate was detected using rabbit to anti-human C3b antibody (1:500) or rabbit to anti-human C4 antibody (1:500), followed by incubation with the secondary antibody HRP-conjugated goat to rabbit anti-IgG (1:1,000). The assays were developed with TMB and the reaction was stopped by the addition of 100 μL 2N sulphuric acid to each well. Absorbance was measured at 450 nm in a PolarStar Omega Spectrophotometer and the OD450 intensity was considered proportional to the C3 or C4 deposition on the wells.

### Statistical analysis

Statistical analysis was carried out using GraphPad Prism version 5. Differences between the groups were assessed using a T-test or One-way ANOVA followed by Dunnett’s multiple comparison test or Newman-Keuls with at least 95% confidence intervals (P values of < 0.05 were considered significant).

## Results

### *F*. *hepatica* NEJ specifically inhibit the Lectin complement pathway

**Fig 1:**
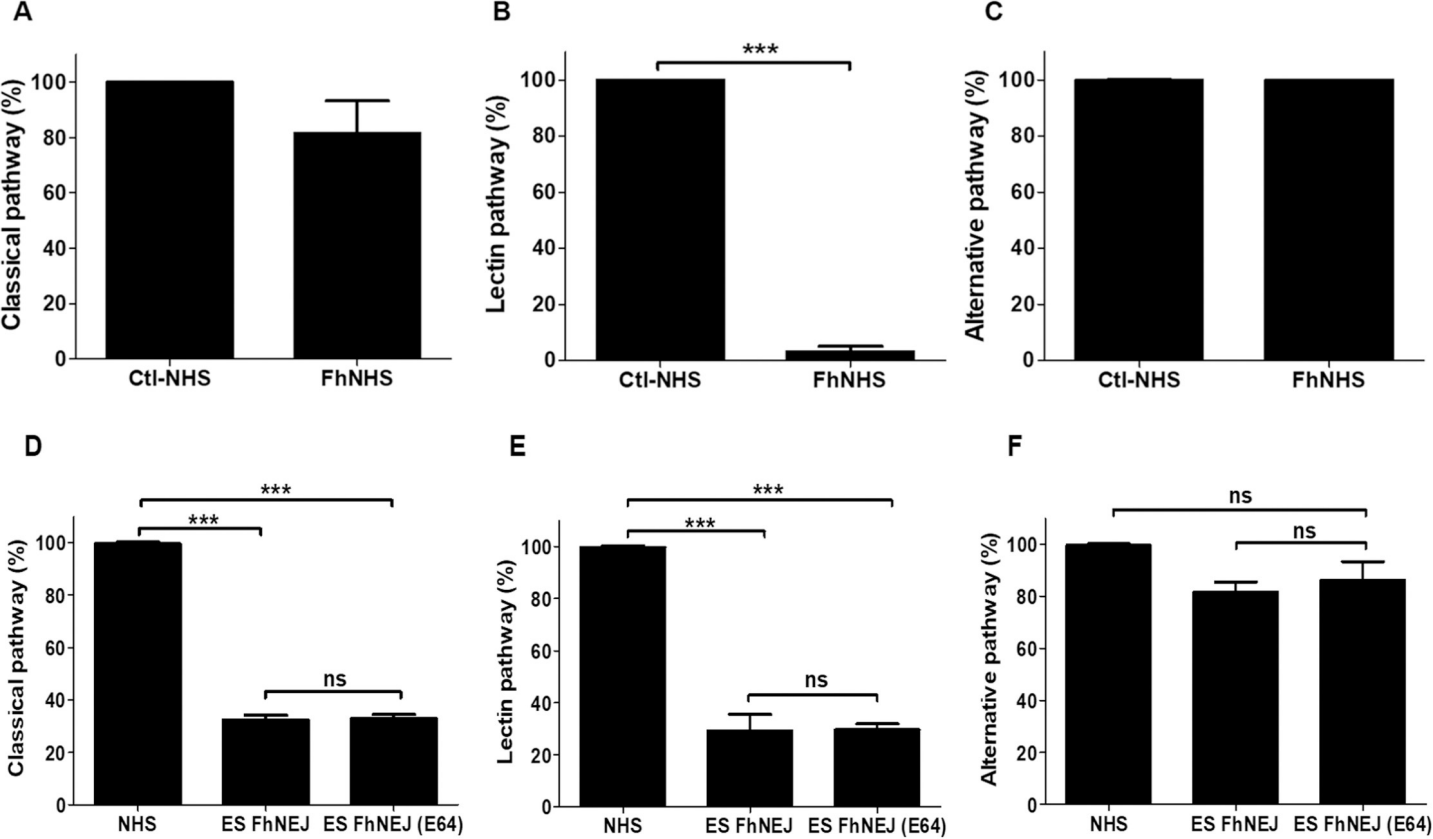
*F*. *hepatica* NEJ specifically inhibit the Lectin pathway of complement. **(A-C)** The activity of the Classical, Lectin and Alternative pathways of complement in normal human sera incubated with live *F*. *hepatica* NEJ (1/μL) (FhNHS) for 1 hr at 37°C was assessed using the Wieslab kit. Data is graphically represented as percentage activity compared to the normal human sera control incubated alone (Ctl-NHS). (**D-F**) The activation of the three pathways was also assessed with NHS incubated with *F*. *hepatica* NEJ ES products alone (ES FhNEJ) or with the cysteine proteinase inhibitor E-64 (FhNEJ (E64)) using the same assay kit. Data is graphically represented as percentage activity compared to the normal human sera (NHS). The experiments were performed in triplicate and the results are represented as means ± standard deviation. Statistical analyses were carried out using a T- test or one-way ANOVA followed by Newman-Keuls. The asterisks indicate significant differences, *** *P* ≤ 0.05. Non-significant results, ns.

During our investigations of how *F*. *hepatica* avoids immune elimination in the early stages of infection we observed that NEJ cultured *in vitro* in 100% NHS for 24 hr were resistant to the killing effects of complement. We then explored the mechanisms by which the NEJ evade complement attack using the Wieslab kit. This commercial 96-well plate assay assesses the three complement pathways, namely Classical, Lectin and Alternative, via the activation with specific ligands (antibody, mannan and LPS, respectively) by measuring the levels of the deposited MAC (C5b-9) on the surface of the well. Remarkably, our results showed that while NHS incubated with *F*. *hepatica* NEJ (FhNHS) retained practically all its activity through the Classical and Alternative pathways, the Lectin pathway was drastically reduced (Fig 1A–1C). We calculated that the incubation of *F*. *hepatica* NEJ with NHS for 1 hr reduced MAC formation via the Lectin pathway >97% when compared to control NHS incubated in the same conditions. Moreover, after the 24 hr incubation the NEJ were completely viable and their locomotion/muscle movements were normal (S2 Fig).

Subsequently we tested the effects of *F*. *hepatica* NEJ ES products on complement activation also using the Wieslab kit. These tests showed that components within this ES preparation could almost completely block the Lectin pathway. Moreover, ES products also have significant activity against the Classical complement pathway (Fig 1D–1F). The effect of the ES products on complement was assayed in the presence and absence of the cysteine peptidase inhibitor, E-64, since we are aware that NEJ secrete both cathepsin L and cathepsin B peptidases. However, this inhibitor did not affect the complement blocking activity of the ES products.

### Live *F*. *hepatica* NEJ prevent the MAC assembling by not allowing the main initiators of the Lectin pathway to bind to their surface

**Fig 2:**
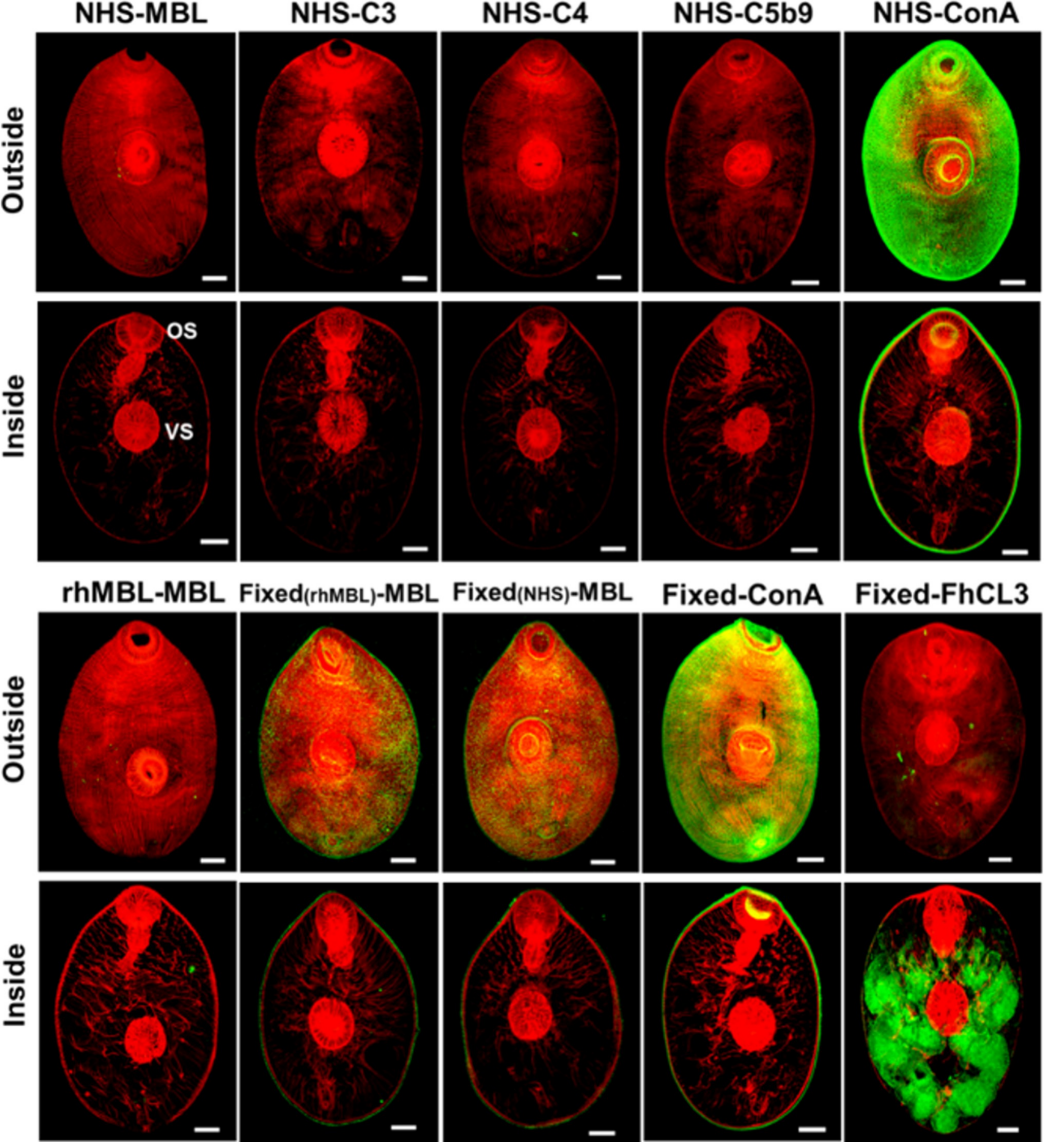
Complement deposition on the *F*. *hepatica* NEJ surface following incubation in human serum. **Top panel**: immunolocalization studies were carried out to assess complement deposition on the surface of whole mount NEJ cultured in normal human sera (NHS) for 1 hr prior to fixation. Once fixed the NEJ were probed with anti-human MBL (1:250), anti-human C3b (1:250), anti-human C4b (1:250), anti-human C5b-9 (MAC, 1:500). As controls, NEJ 24 hr were also probed with the mannose-binding lectin, concanavalin A (ConA, 1:200). **Bottom panel**: NEJ were fixed and then incubated with recombinant human MBL (rhMBL, 2 μg/mL) or NHS for 1 hr before being probed with anti-human MBL (1:250). As controls, NEJ were fixed and probed with ConA (1:200), which stains the surface of the NEJ, or with rabbit anti-*F*. *hepatica* cathepsin L3 (FhCL3, 1:500), which highlights the peptidase in the bifurcated gut. All samples were analysed by confocal laser microscopy represented by green fluorescence (FITC staining) and counter-stained with phalloidin-tetramethylrhodamine isothiocyanate (TRITC) for visualisation of the NEJ musculature (red fluorescence). The profile of immunolocalization is shown on two planes; on the surface of the NEJ (Outside) and internally (Inside). OS, oral sucker. VS, ventral sucker. Scale bars, 25 μM.

To investigate how the parasite blocks the Lectin complement pathway we first carried out immunolabelling experiments on fixed *F*. *hepatica* NEJ following their incubation in NHS. No significant fluorescent labelling could be detected on the surface of these NEJ parasites when we probed them with commercially available antibodies against various salient complement components, MBL, C3b, C4b and MAC (C5b-9); however, a strong green fluorescence signal could be detected when the larvae were probed with another lectin, the concanavalin A (ConA), which binds to mannosylated sugars (Fig 2).

Live *F*. *hepatica* NEJ were also cultured with rhMBL, the molecule responsible for the initiation of the Lectin pathway, and then probed with anti-hMBL. No fluorescence could be observed; however, by marked contrast, when NEJ were first fixed and subsequently exposed to either rhMBL or NHS, a significant amount of hMBL could be detected on the surface of the NEJ (Fig 2). Similarly, fixed NEJ probed with anti-C4b have also showed substantial fluorescence signal on the surface. However, even in these conditions, C3b and C5b-9 do not deposit on the NEJ’s surface, as indicated by the absence of green fluorescent signal in these specimens (S3 Fig). Control experiments show that strong immunofluorescent signals were present in the gut and on the surface of fixed *F*. *hepatica* NEJ probed with anti-FhCL3 and ConA, respectively (Fig 2). These results suggest that while the surface of live NEJ is refractory to hMBL binding, the surface of fixed larvae is not. Moreover, the presence of C4b on the surface of fixed NEJ exposed to NHS, but not on the live ones, indicates that these larvae secrete molecules that actively prevent the formation of essential complement intermediates on their surface.

**Fig 3:**
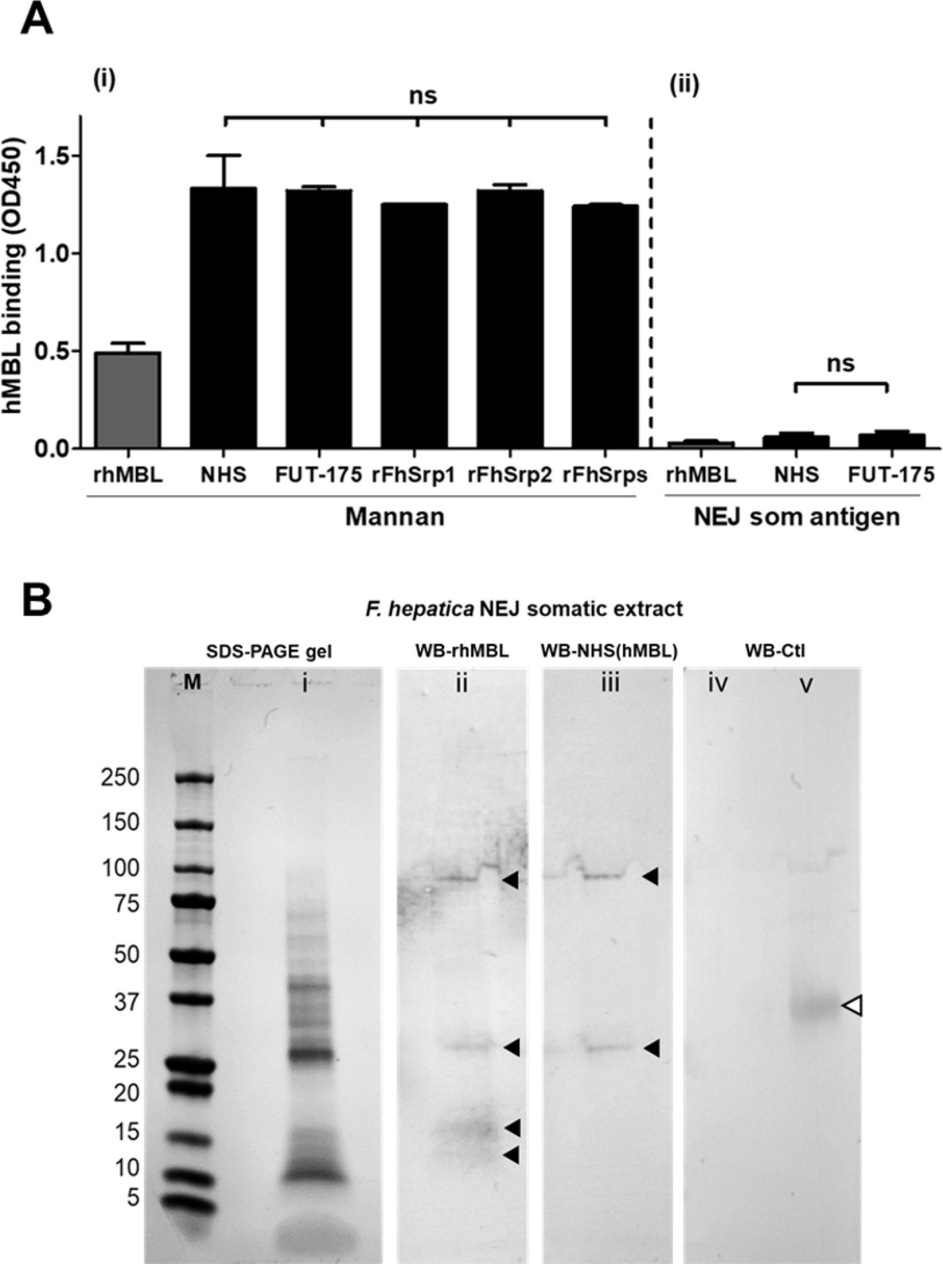
Recognition of glycans in the *F*. *hepatica* NEJ somatic extract by human mannose binding lectin. **(A)** The ability of recombinant human mannan binding lectin (rhMBL) or native human mannan binding lectin in NHS (NHS) to bind to ELISA plates coated with **(i)** mannan from *S*. *cerevisiae* or **(ii)** NEJ somatic extract-coated (NEJ som antigens). The inhibition of the broad-spectrum serine protease inhibitor FUT-175 (100 μM), or of 1 μM recombinant *F*. *hepatica* serpins (rFhSrp1, rFhSrp2, or combined rFhSrps) was assessed and compared relative to the binding observed with the NHS alone. The experiments were performed in triplicate and the results represented as means ± standard deviation on independent analyses. Statistical analysis was carried out using One-way ANOVA with Dunnett multiple comparison. Non-significant results, ns. **(B)** Binding of rhMBL or native hMBL in NHS to *F*. *hepatica* NEJ somatic extract by Western blot analysis (WB). NEJ somatic extract was resolved in a 4–20% SDS-PAGE **(i)** and electro-transferred onto PVDF membranes. The membranes were probed with **(ii)** rhMBL (2 μg/mL) or **(iii)** NHS (1:25) as source of hMBL. Additionally, as controls, **(iv)** NEJ extract and **(v)** rhMBL (white arrow, ~34 kDa) were probed only with the secondary rabbit anti-human MBL-HRP conjugated antibody (1:4,000). M, molecular weight in kilodaltons.

We next investigated if hMBL, recombinant or native, could bind to glycoconjugates in *F*. *hepatica* NEJ somatic extracts using ELISA and transfer blots assays (Fig 3). While both rhMBL and native hMBL (NHS) were demonstrated to bind to wells coated with mannan from *S*. *cerevisiae* (Fig 3A, i), no significant binding could be detected in wells coated with the NEJ somatic extract (Fig 3A, ii). However, when the *F*. *hepatica* NEJ somatic extract was transferred onto PVDF membrane and probed with either recombinant or native hMBL, distinct bands could be observed, indicating that hMBL binds specific glycosylated proteins in the parasite extract (Fig 3B). As a control, we also tested if serine protease inhibitors (rFhSrps or FUT-175) could interfere with MBL binding. Our results demonstrate that neither of the inhibitors reduce MBL ability to bind to mannan or NEJ extract.

### rFhSrp1 and rFhSrp2 inhibit the Lectin pathway but not the classical or alternative pathways

**Fig 4:**
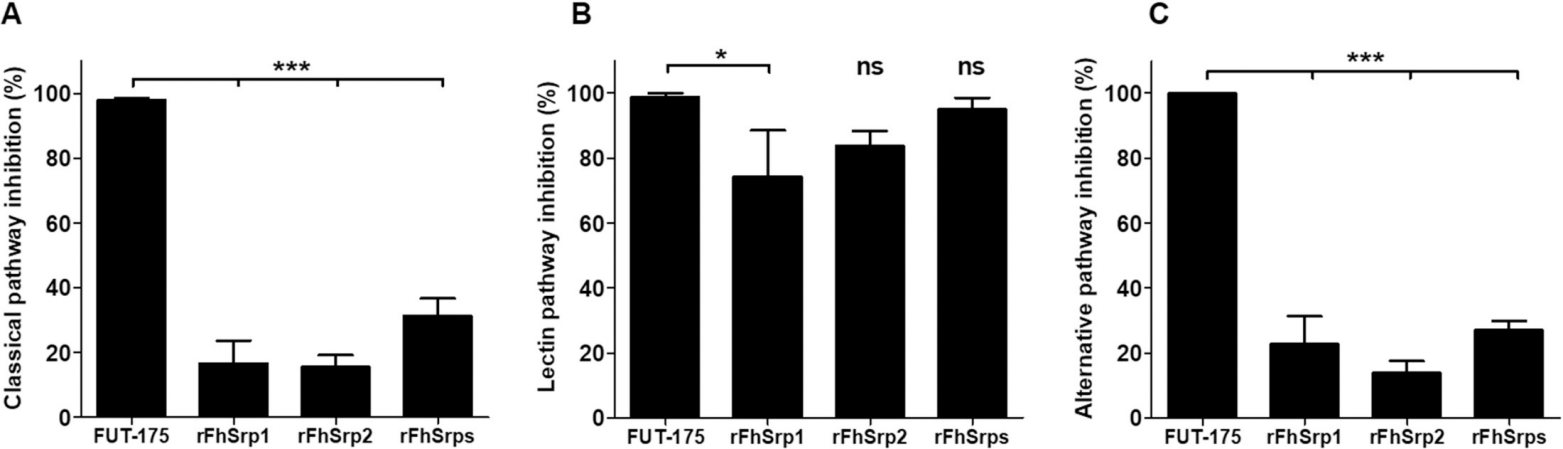
Specific inhibition of the Lectin pathway of complement by *F*. *hepatica* serine protease inhibitors, rFhSrp1 and rFhSrp2. The activity of the three complement pathways, **(A)** Classical, **(B)** Lectin and **(C)** Alternative, in normal human sera was assessed using the Wieslab kit in the presence of rFhSrp1 (1 μM), rFhSrp2 (1 μM) or a combination of the two recombinant serpins, rFhSrps (1 μM). The serine protease inhibitor FUT-175 (100 μM) was used as a positive control. The data from experiments performed in triplicate are graphically represented as percentage inhibition compared to normal human sera alone and the results are presented as means ± standard deviation. Statistical analysis was carried out using One-way ANOVA compared to the positive control. The asterisks indicate significant differences, *P ≤ 0.05, *** *P* ≤ 0.001, and ns are non-significant results.

We have recently shown that during their development in the mammalian host *F*. *hepatica* expresses and temporally regulates a family of serpins. We produced functionally active recombinant forms of two members of this family, rFhSrp1 and rFhSrp2, which were observed to localise to the surface of the NEJ, but are also highly excreted/secreted by this parasite lifecycle stage [24, 31]. Given that the cascade of events that mediate complement activation involves many different serine proteases [32], we examined the ability of rFhSrp1 and rFhSrp2 to block individual complement pathways using the Wieslab kit. Firstly, the results show that the broad-spectrum serine protease inhibitor, FUT-175 (100 μM), blocked all three complement pathways by >95%. Secondly, and by contrast, we found that rFhSrp1 and rFhSrp2 (1 μM) inhibited the activation of the Lectin pathway by 74% and 84%, respectively, and this effect was further increased (>95%) when the two serpins (FhSrps) were combined in the assays (Fig 4B). Such inhibition of the Lectin cascade was proportional to that observed with the live NEJ or the positive control, FUT-175 (*P* ≤ 0.05) (Fig 4). The serpins also showed some blocking of the classical and alternative pathways, but this was always below 25%, even when the two inhibitors were combined (Fig 4A and 4C).

### rFhSrp1 and rFhSrp2 bind to serum-derived MBL-associated serine proteases (MASPs) and inhibit their activity

**Fig 5:**
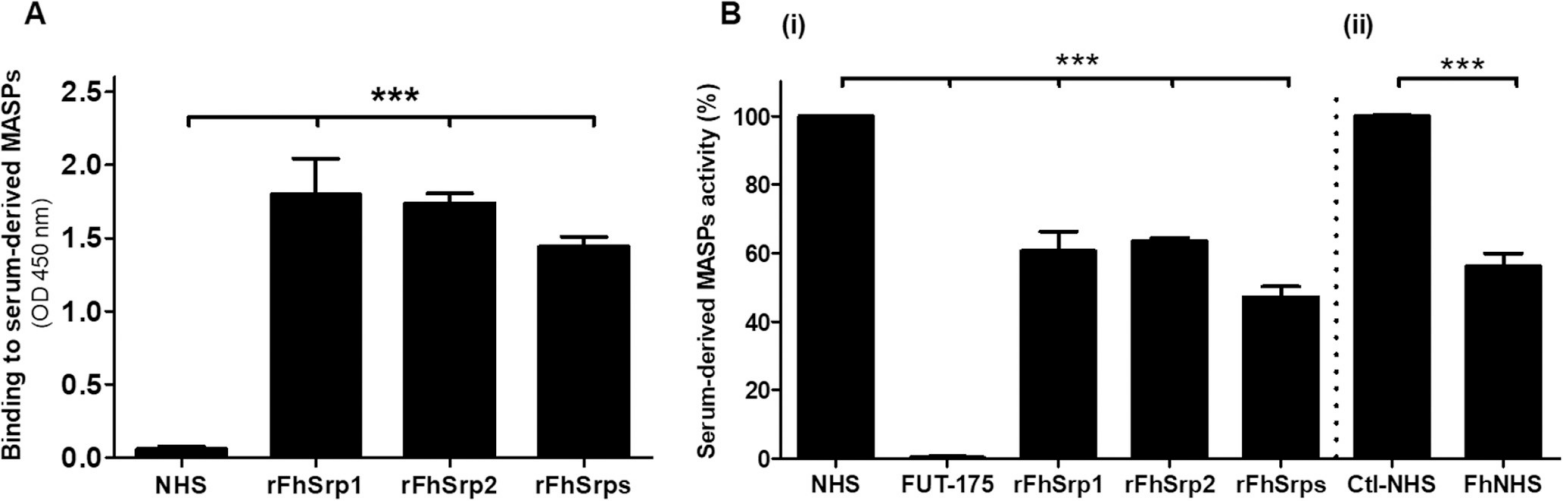
*In vitro* binding and inhibitory effects of *F*. *hepatica* rFhSrp1 and rFhSrp2 on serum-derived MASPs. **(A)** Graphical representation of the binding of rFhSrp1, rFhSrp2 or combined serpins (rFhSrps) to serum-derived MASPs evaluated by ELISA, relative to the control normal human sera (NHS). **(Bi)** The inhibitory effect on serum-derived MASPs by the recombinant serpins, rFhSrp1 (1 μM), rFhSrp2 (1 μM) or rFhSrps (1 μM). The serine protease inhibitor FUT-175 (100 μM) was used as a positive control. MASPs activity was graphically represented as percentage activity compared to the activity in NHS alone set as 100% activity. **(Bii)** The MASPs activity in NHS in which *F*. *hepatica* NEJ were cultured at 37°C for 1 hr (FhNHS) was graphically represented as percentage activity compared to the normal human sera incubated alone, as control, set as 100% activity (Ctl-NHS). The experiments were performed in triplicate and the results are represented as means ± standard deviation on independent assays. Statistical analysis was carried out using One-way ANOVA with Dunnett multiple comparison and T-tests compared to the NHS control. The asterisks indicate significant differences, *** *P* ≤ 0.05.

MBL-associated serine proteases (MASP-1 and MASP-2) are exclusive to the Lectin complement pathway [33]. They form complexes with the pattern recognition molecules of the Lectin pathway (i.e. MBL, ficolins and collectins) in the circulation. When these complexes bind to a suitable pattern of glycans expressed on the surface of pathogens, the MASPs become activated. MASP-1 activates zymogen MASP-2 and both cleave C2 and C4 to form the C3-convertase (C3c) required for proper Lectin pathway activation [23, 34]. We investigated the ability of *F*. *hepatica* serpins to specifically bind and inhibit MASPs activity using NHS as a source of native MASPs. We first designed an ELISA based assay that employed anti-serpin antibodies to assess the ability of serpins to bind MASPs. Our data showed that the *F*. *hepatica* serpins, rFhSrp1 and rFhSrp2, are capable of binding serum-derived MASPs and, at 1 μM, both displayed similar affinity (Fig 5A). The OD values of all the samples containing serpins were significantly higher (*P* ≤ 0.05) than those obtained for the control NHS samples without serpins (Fig 5A).

Next, to assess if the binding of rFhSrp1 and rFhSrp2 to serum-derived MASPs reflects a mechanism of inhibition, we assayed the activity of native-bound MASPs in the presence and absence of the serpins using the fluorogenic substrate Z-Ile-Glu-Gly-Arg-AMC. Both rFhSrp1 and rFhSrp2, at 1 μM, significantly reduced the activity of the serum-derived MASPs by ~40% (Fig 5B, i). Interestingly, the combination of the two serpins (rFhSrps) resulted in an even more efficient inhibition, decreasing the enzymatic activity by ~50%. As expected, total inhibition of native MASPs activity was achieved with the broad-spectrum serine protease inhibitor FUT-175 at 100 μM, which is the recommended concentration for inhibition of the complement response via any complement pathway (Fig 5B, i). Finally, we found that MASPs activity in NHS pre-incubated for 1 hr with *F*. *hepatica* NEJ was reduced by ~45%, compared to the untreated control sera (Fig 5B, ii).

### The recombinant catalytic domain of MASPs, crMASP-1 and crMASP-2, are inhibited by *F*. *hepatica* serpins

Having shown that the recombinant serpins inhibit native MASPs in NHS we proceeded to investigate if such inhibition resulted from the interaction of the serpins with the catalytic domain of MASP-1 and/ or MASP-2. For this analysis, we used recombinant active forms of the catalytic fragment of the crMASP-1 (CCP1-CCP2-SP) and crMASP-2 (CCP1-CCP2-SP), which consist of two complement control protein modules (CCP1-CCP2) and a chymotrypsin-like serine protease (SP) domain [35]. Previous studies have shown that these domains efficiently cleave the proteins within the complement cascade, specifically crMASP-1 (CCP1-CCP2-SP) cleaves C2 and crMASP-2 (CCP1-CCP2-SP) cleaves C2 and C4 [25].

**Fig 6:**
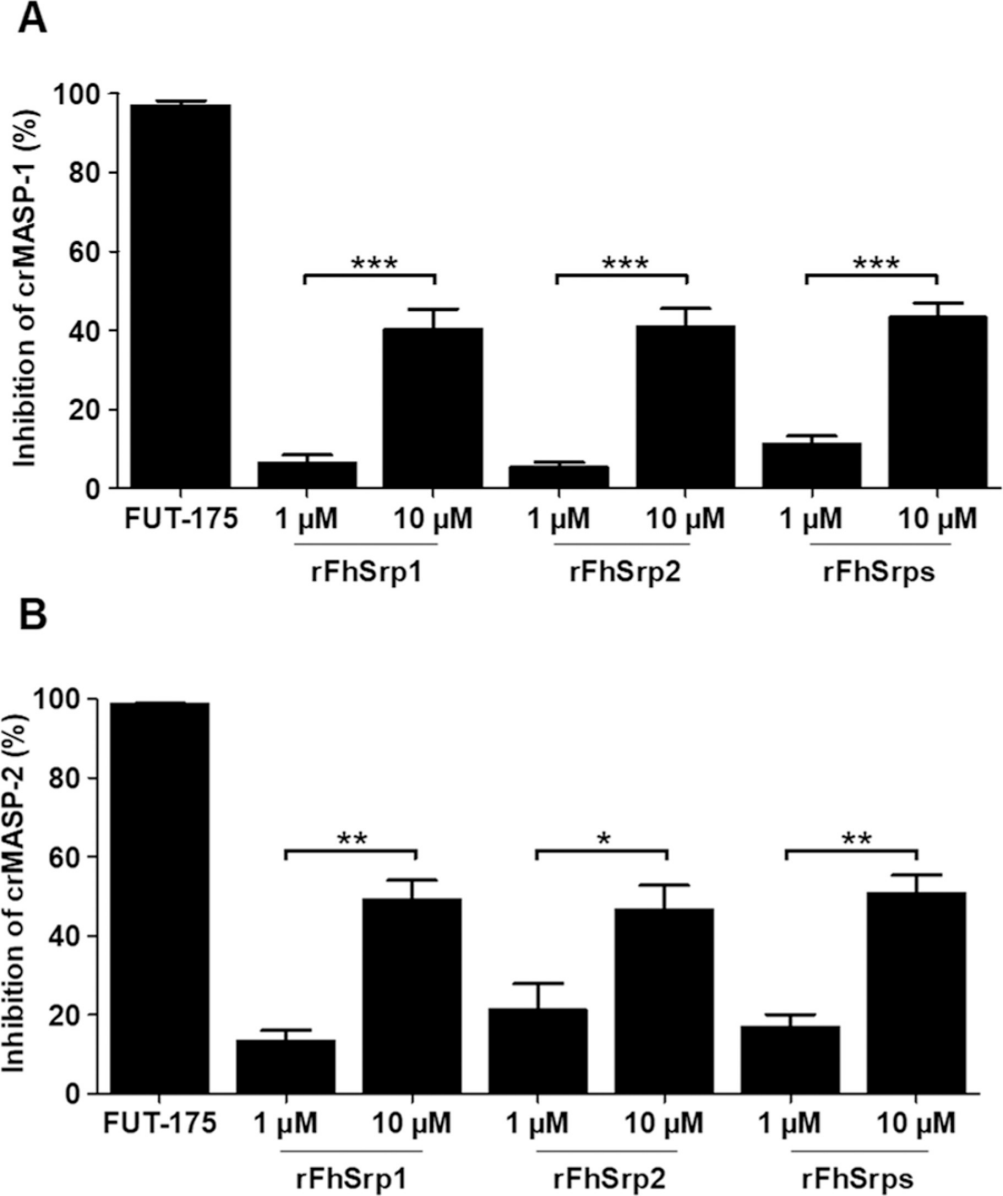
Inhibition of recombinant crMASP-1 and crMASP-2 by rFhSrp1 and rFhSrp2. The activity of the recombinant catalytic domain of **(A)** crMASP-1 (0.1 μM) and **(B)** crMASP-2 (0.2 μM) was assayed alone or in the presence of 1 and 10 μM of rFhSrp1, rFhSrp2 or combined rFhSrps. The broad-spectrum serine protease inhibitor FUT-175 (100 μM) was used as a positive control. The experiments were performed in triplicate and the enzymatic activity in each condition is presented as means ± * P< 0.05, **P< 0.01.

Surprisingly, the activity of the recombinant crMASP-1 (0.1 μM) or crMASP2 (0.2 μM) was only marginally affected by either *F*. *hepatica* serpins at 1 μM. However, when rFhSrp1 and rFhSrp2 were used at 10 μM in the assays, ~40% and ~50% of the activity of crMASP-1 and crMASP-2 was abrogated, respectively (Fig 6A and 6B). At 10 μM, both serpins were more effective at inhibiting crMASP-2 than crMASP-1, whilst FUT-175 completely abrogated the enzymatic activity of both catalytic domains when used at the manufacturer’s recommended concentration of 100 μM. These results suggest that the interaction of *F*. *hepatica* serpins with the small recombinant catalytic domain of MASPs is not as efficient as their interaction with the native proteinases.

### rFhSrp1 and rFhSrp2 reduce *in vitro* C3 and C4 deposition via the Lectin complement pathway

**Fig 7:**
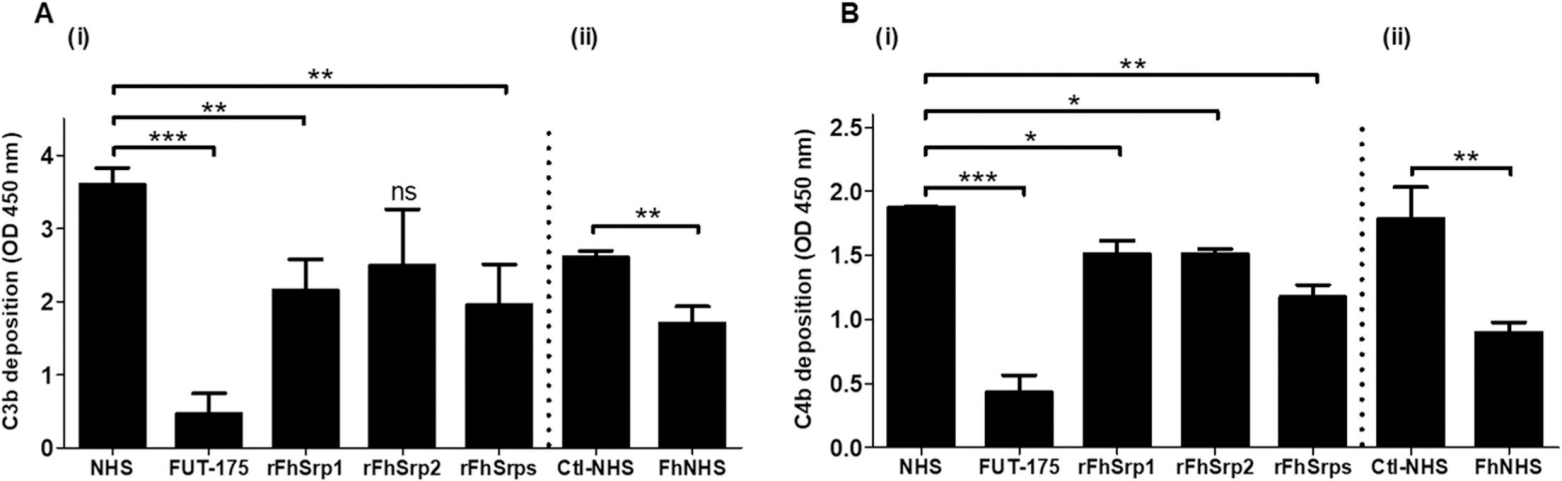
*F*. *hepatica* NEJ, rFhSrp1 and rFhSrp2 reduce *in vitro* C3 and C4 deposition mediated by the Lectin complement pathway. Graphical representation of the deposition of **(A)** C3b and **(B)** C4b on the surface of mannan-coated plates in the presence of **(i)** recombinant serpins rFhSrp1 (1 μM), rFhSrp2 (1 μM) or two serpins combined, rFhSrps (1 μM). The broad-spectrum serine protease inhibitor FUT-175 (100 μM) was used as a positive control. Complement deposition was graphically represented as OD450 values and compared to the deposition obtained with NHS alone. **(ii)** Deposition caused by normal human serum (NHS) in which *F*. *hepatica* NEJ were cultured at 37°C for 1 hr (FhNHS) relative to normal human sera incubated alone as control (Ctl-NHS). The experiments were performed in triplicate and the results represented as means ± standard deviation on independent analyses. Statistical analysis was carried out using One-way ANOVA with Dunnett multiple comparison and T-tests compared to the NHS control. The asterisks indicate significant differences, *P ≤ 0.05, **P ≤ 0.01, *** *P* ≤ 0.001, and ns are non-significant results.

We investigated the effect of rFhSrp1 and rFhSrp2 on MBL/MASPs-mediated cleavage of C3 and C4 and their subsequent deposition on targeted surfaces. Using an ELISA-based approach to quantify the complement deposition, we showed that rFhSrp1 significantly reduced both C3b and C4b deposition (*P* ≤ 0.05), whilst the rFhSrp2 was more efficient at reducing the C4 deposition (Fig 7A and 7B). When a combination of the two recombinant serpins were used, FhSrps, their inhibitory effect towards sera derived-MASPs, and consequently C3b and C4b deposition, are potentiated (Fig 7A and 7B). rFhSrps reduced the C3b and C4b deposition by ~50 and ~35%, respectively, whereas FUT-175 (100 μM) inhibited the deposition of both by >80% (*P* ≤ 0.05).

Significant inhibition of C3b and C4b deposition was also observed using NHS that was exposed to live *F*. *hepatica* NEJ (~30 and ~50%, respectively (*P* ≤ 0.05), compared to the untreated control sera (Fig 7A and 7B, ii).

## Discussion

Activation of the complement system is critical to many biological processes, including phagocytosis, lysis, and inflammation, and is pivotal for the regulation of adaptive immune responses that elicit antibodies to T-cell dependent and independent antigens [2, 36]. Its activation via any of the three pathways, Classical, Lectin or Alternative, leads to deposition of complement factors on target organisms (opsonisation) and culminates in the assembly of the lytic membrane attack complex (MAC) resulting in phagocytosis and lysis, respectively [4]. Complement responses exert significant pressure on pathogens, particularly during the early invasive stages, and are critical to prevent further dissemination of the infectious agent [37, 38].

In the case of the liver fluke *F*. *hepatica*, as the adult fluke hides inside the bile duct, the complement system would be expected to rapidly direct its focus towards the early NEJ stage as they invade and migrate through the host’s intestinal tissues to establish infection. Although it is known that NEJ parasites have evolved mechanisms to counteract both innate and adaptive responses of the mammalian host [19, 20], at present, it is not understood how they avoid the host complement attack. In this study, we found that live *F*. *hepatica* NEJ survive undamaged when incubated with normal human serum (S2 Fig). This was a surprising result, especially given that a recent characterization of glycans associated with the tegumental surface of NEJ revealed an abundance of highly mannosylated sugars that would be expected to make them susceptible to the Lectin complement cascade [22, 39, 40]. Indeed, our immunocytochemical analysis of live *F*. *hepatica* NEJ demonstrated intense fluorescent signals when these were probed with the lectin concanavalin A (ConA) confirming the presence of exposed mannosylated sugars on the parasite surface.

Complement activation via the Lectin pathway is initiated when MBLs bind to mannosylated glycans on the surface of pathogens [23, 41]. However, no significant binding of hMBL was observed on the NEJ surface after incubation with NHS or recombinant hMBL, explaining why the Lectin pathway is not initiated by the parasite. This also clarifies why downstream complement factors such as C3b, C4b and MAC (C5b-9) were not observed bound to the surface of live *F*. *hepatica* NEJ that were incubated in NHS. Furthermore, C3b and C5b-9 were not detected on the surface of dead NEJ exposed to NHS, in contrast to that observed for MBL and C4b, suggesting that the parasite actively secretes complement inhibitory molecules.

Early ultrastructural studies of developing *F*. *hepatica* parasites showed that their tegument is covered by a ‘fuzzy’ glycocalyx that varied in composition according to the environment in which the parasites were obtained, i.e., intestine, liver or bile duct [42, 43]. Although the Lectin pathway was not discovered until years later, Hanna [43] had proposed that the continual shedding of the NEJ glycocalyx, following exposure to immune sheep serum, represented a pivotal strategy for parasite survival by preventing complement binding and activation. Subsequently, studies by Davies and Goose [44] described their inability to detect C3 deposition on the surface of live NEJ incubated in immune rat serum or on flukes collected from the peritoneal cavity of sensitized rats. Although we did not detect hMBL bound on the surface of live NEJ incubated in NHS, we did observe binding of hMBL to parasites if they were first fixed and then exposed to NHS. Moreover, recombinant hMBL recognized specific bands in soluble extracts of NEJ that were electro-transferred to PVDF membranes. Therefore, we propose that NEJ possess molecules containing hMBL-glycan epitopes, but their topology on the surface of live NEJ does not facilitate their recognition and the correct assembly of hMBL to initiate the Lectin pathway. If some MBL bound to the surface this could be discarded by the continual sloughing of the glycocalyx, suggested by Hanna [43], or this mechanism may not allow activators of the complement response to bind with sufficient affinity, or for sufficient time, on the surface to initiate the specific cascades.

The importance of preventing the activation of the Lectin complement pathway by *F*. *hepatica* NEJ is further highlighted by our studies showing that they possess a secondary, or back-up, mechanism to regulate this cascade. We discovered that pre-incubation of NHS with live *F*. *hepatica* NEJ prevents the assembly of MAC on the surface of mannose-coated wells of the Wieslab kit, further indicating that the NEJ release factors that specifically block the activation of the Lectin complement pathway. Serpins expressed by vertebrates are commonly involved in the regulation of complex cascades such as those for blood coagulation and complement activation [45]. Our recent immunolocalization studies show that *F*. *hepatica* NEJ express FhSrp1 and FhSrp2 on their surface [24]. In addition, proteomic analysis reveals that these serpins are released within the NEJ’s secretions, consistent with the presence of secretory signal sequences on these proteins [31, 46]. Here, we showed that the NEJ ES products have a profound blocking effect on the ability of NHS to activate the Lectin pathway in the Wieslab kit, and this inhibition could be replicated using recombinant forms of the FhSrp1 and FhSrp2 at low concentrations (1 μM).

Because *F*. *hepatica* NEJ do not excrete/secrete serine proteases, we proposed that FhSrp1 and FhSrp2 were designed to inhibit host serine proteases and, therefore, would play key roles in the parasite-host interplay [24]. As the Lectin complement pathway uniquely relies on the activity of MBL-associated serine proteases, namely MASP-1 and MASP-2 [25, 33, 34, 47], we were drawn to investigate the effect of *F*. *hepatica* serpins on these proteases. Both rFhSrp1 and rFhSrp2 bound and significantly inhibited the activity of native MASPs in NHS by ~40% at 1 μM, leading to a proportional reduction of the *in vitro* deposition of C3b and C4b on mannose-coated plates. The less potent inhibition of the rFhSrp1 and rFhSrp2 on the recombinant forms of MASP-1 and MASP-2 (crMASP-1 and crMASP-2) could represent a concentration effect of MASPs in NHS in relation to the recombinant versions used in the *in vitro* assays. However, it is worth noting that these synthetic forms represent only the central catalytic fragment of the enzymes [25, 35] and, as such, may exhibit different binding characteristics to native MASPs which undergo a conformational change following binding to MBL [45].

Although serpins of different parasites have been suggested to be involved in the regulation of various homeostatic processes within the host [48], their role as complement inhibitors are not well characterized. Interestingly, Verma et al. [1], obtained comparable results to our study with a *Leishmania donovani* serine protease inhibitor (rLdSPI2) which, at 50 μM, inhibited MASP-2 activity by ~60%. Mika et al. [49], found that two scabies mite serpins, termed SMSB3 and SMSB4, inhibited all three pathways of the human complement system, although the authors did not observe complex formation between either of these mite serpins and MASPs [49]. However, the fact that serine protease inhibitors of *F*. *hepatica* and *L*. *donovani* target MASPs’ activity may be the first indication of a common strategy of complement evasion amongst both helminth and protozoan parasitic organisms [1, 3, 48].

Complement evasion strategies have been reviewed for several different helminth parasites, and mainly involve the acquisition or expression of host-like complement regulators on their surface [6, 50–52]. Avoidance of complement attack by schistosome parasites, flatworms related to *F*. *hepatica* that also migrate through mammalian host tissues at early stages and feed on blood as adults, is thought to be intricately linked to their decades-long survival within the host. They exploit several overlapping strategies to prevent damage by complement, including replacement of their tegumental surface, appropriation of host complement receptors (e.g. decay accelerating factor, DAF), and expression of surface molecules like paramyosin and CD59-like proteins, which avoid C3, C8 and C9 components binding to stop their activation or assembly into functional complexes that could lead to parasite damage [9, 11, 52]. While these evasion strategies prevent damage and elimination, they may also contribute to creating an immunological environment favourable for the parasite invasion and establishment within the host, as the complement response also initiates the innate and adaptive immune responses and stimulates pro-inflammatory responses [38].

Although helminth parasites are often coated with glycans [22, 53, 54], up to now, few studies have focused on the complement response via the Lectin pathway in the context of these infections. In the present study, we show that live *F*. *hepatica* NEJ escape the innate host response via the Lectin complement pathway by avoiding the binding of the main recognition molecule, MBL. In addition, the NEJ secrete and express serpins on their surface that interfere with MASPs activity and thereby halt the cascade of reactions that lead to complement activation and the formation of lytic compounds. In addition, as MASP-1 is capable of initiating the Lectin complement pathway by itself, the ability of *F*. *hepatica* serpins to inhibit this enzyme could explain the drastic effect both the recombinant inhibitors and the live NEJ have on this cascade [34]. This mechanism has not been described before for any other helminth parasite. However, it may be only a part of the overall strategy by which *F*. *hepatica* NEJ block complement-mediated killing.

As shown here, molecules within the NEJ ES products also significantly inhibit the Classical complement pathway and, although the identity of these is unknown, they do not appear to be cathepsin-like cysteine proteinases. As the Classical pathway is initiated by antigen-antibody complexes this cascade could play a significant role in cases of re-exposure to the parasite. The NEJ’s ability to prevent complement attack via this pathway could be important for resistance during *F*. *hepatica* re-infections. Unveiling this mechanism will involve future studies of the key NEJ ES molecules that interact with the multitude of complement components.

**Fig 8:**
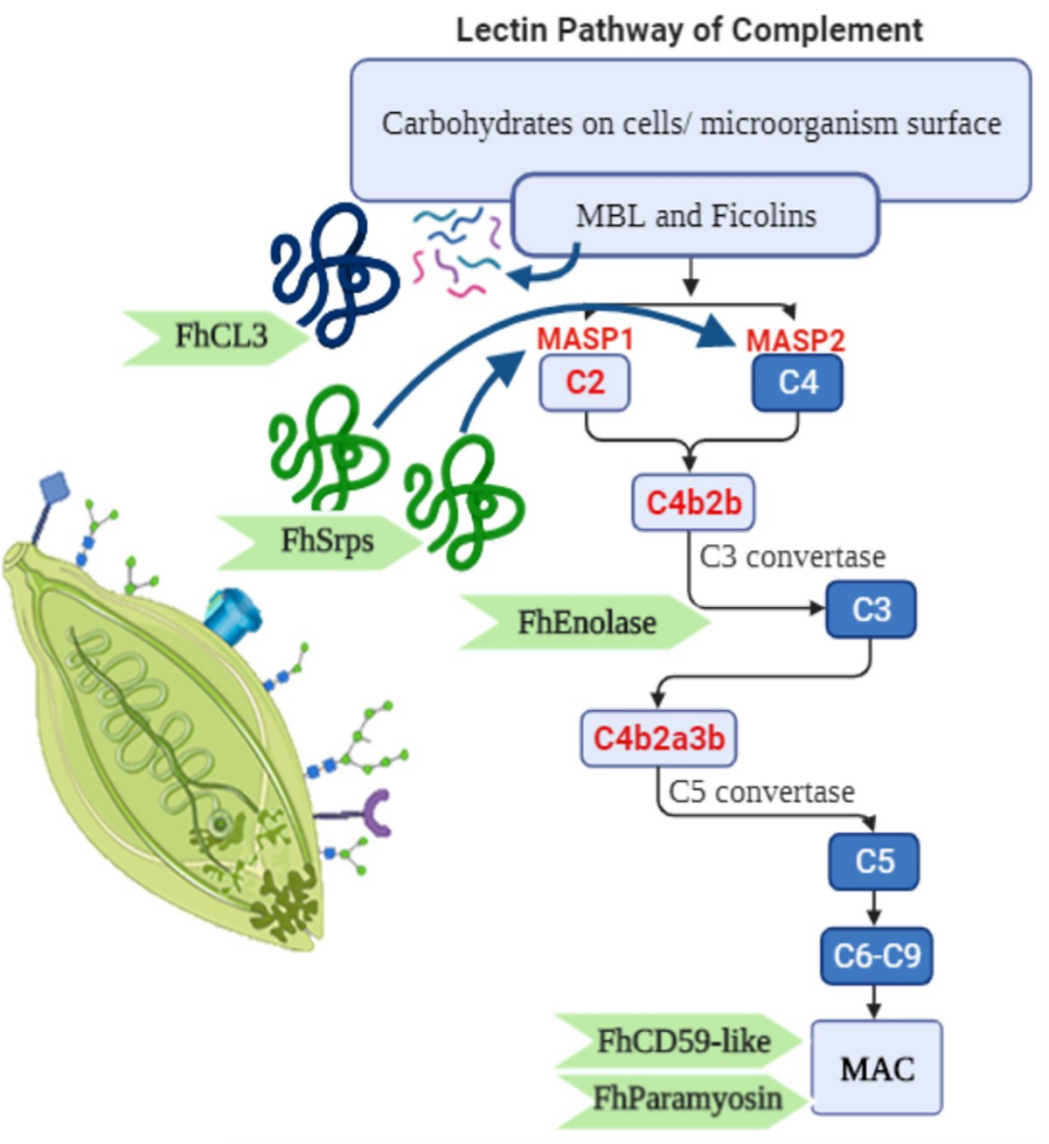
Schematic of the putative mechanisms *F*. *hepatica* NEJ employ to disrupt the complement pathway. The possible *F*. *hepatica* targets within the Lectin pathway are highlighted in light blue. Key serine proteases within the Lectin cascade (in red) are all potential targets of *F*. *hepatica* serpins (FhSrps). The highly secreted cathepsin L3 protease (FhCL3) is known to have a strong collagenolytic activity and might impair the Lectin pathway of complement by digesting the collagen-like domain of the mannose binding lectin (MBL) and/ or ficolins. The proteins FhCD59-like, paramyosin and enolase are found in the tegumental surface of *F*. *hepatica* life stages and may act as regulators of complement activity (RCA). Images built using biorender https://app.biorender.com/illustrations/ and smart server https://smart.servier.com/.

In conclusion, the present study shows that *F*. *hepatica* NEJ survive undamaged during the processes of invasion and migration through host tissues by preventing activation of the Lectin complement pathway. This inhibition appears to be achieved by multiple and overlapping mechanisms, namely expression of a glycosylated surface refractory to MBL, the main recognition molecule of this cascade, and surface expression and secretion of serpins that halt MASPs’ activity, ultimately limiting the formation of lytic complement molecules on the parasite. Additional strategies used by *F*. *hepatica* to evade, disarm or inhibit the complement pathways could include variation of the tegmental surface antigens [52], and the expression of proteolytic enzymes [55], complement-binding molecules such as paramyosin and enolase [9, 56–58], and/or host-like receptors such as CD59-like proteins [59, 60]. Several of these molecules are expressed by all the *F*. *hepatica* developmental stages within the mammalian host [28, 46, 61, 62], and may function in conjunction to stop host complement attack and facilitate parasite establishment [38] (Fig 8). Further elucidation of how *F*. *hepatica* and other helminths evade complement attack is the next step for the discovery of novel anti-parasite interventions.

## Supporting information

S1 Fig Screening substrates to assess crMASP-1 and crMASP-2 enzymatic activity. The activity of recombinant **(A)** crMASP-1 (0.1 μM) and **(B)** crMASP-2 (0.2 μM) was assayed with different fluorogenic substrates, namely Z-Gly-Pro-Arg-AMC (GPR, 40 μM; Bachem, UK), Z-Leu-Arg-AMC (LR, 40 μM; Bachem, UK), Z-Phe-Arg-AMC (FR, 40 μM; Bachem, UK), Z-Val-Ile-Arg-AMC (VIR, 40 μM; Bachem, UK), Z-Ile-Glu-Gly-Arg- AMC (IEGR, 40 μM; Bachem, UK). The proteolytic reactions were performed in TBS-Ca^+2^ (150 mM NaCl, 50 mM Tris, 20 mM CaCl_2_, 0.05% Tween-20 (*v/v*), pH 7.8) and measured continuously for up to 1 hr at 37°C, as relative fluorescent units (RFU) in a PolarStar Omega Spectrophotometer (BMG LabTech, UK). All assays were carried out in triplicate and are represented as means ± standard deviation.
(DOCX)

S2 Fig *F*. *hepatica* NEJ survive incubation in Normal Human Serum. **(A)** NEJs 3 hr post-excystment in PBS. **(B)** NEJ 24 hr post-excystment which were kept incubated in RPMI medium, at 37°C with 5% CO_2_. **(C)** NEJ 24 hr post-excystment which incubated in 100% Normal Human serum (NHS), at 37°C with 5% CO_2_. Images were made using a light microscope (25x magnification). Scale bars, 10 mm.
(DOCX)

S3 Fig Complement deposition on the surface of dead *F*. *hepatica* NEJ following incubation in human serum. Immunolocalization studies were carried out to assess complement deposition on the surface of whole mount NEJ cultured in RPMI medium, fixed and then incubated with NHS for 1 hr before being probed with anti-human MBL (1:250), anti-human C3b (1:250), anti-human C4b (1:250) or anti-human C5b-9 (MAC, 1:500). All samples were analysed by confocal laser microscopy represented by green fluorescence (FITC staining) and counter-stained with phalloidin-tetramethylrhodamine isothiocyanate (TRITC) for visualisation of the NEJ musculature (red fluorescence). The profile of immunolocalization is shown on two planes; on the surface of the NEJ (Outside) and internally (Inside). OS, oral sucker. VS, ventral sucker. Scale bars, 25 μM.
(DOCX)
