# Supplementary material for: Fasciola hepatica is refractory to complement killing by preventing attachment of mannose binding lectin (MBL) and inhibiting MBL-associated serine proteases (MASPs) with serpins
Source: PLoS Pathog. 2022 Jan 10;18(1):e1010226. doi: 10.1371/journal.ppat.1010226 (PMC8782513; doi:10.1371/journal.ppat.1010226)
Supplement: S1 Fig — The activity of recombinant (A) crMASP-1 (0.1 μM) and (B) crMASP-2 (0.2 μM) was assayed with different fluorogenic substrates, namely Z-Gly-Pro-Arg-AMC (GPR, 40 μM; Bachem, UK), Z-Leu-Arg-AMC (LR, 40 μM; Bachem, UK), Z-Phe-Arg-AMC (FR, 40 μM; Bachem, UK), Z-Val-Ile-Arg-AMC (VIR, 40 μM; Bachem, UK), Z-Ile-Glu-Gly-Arg- AMC (IEGR, 40 μM; Bachem, UK). The proteolytic reactions were performed in TBS-Ca+2 (150 mM NaCl, 50 mM Tris, 20 mM CaCl2, 0.05% Tween-20 (v/v), pH 7.8) and measured continuously for up to 1 hr at 37°C, as relative fluorescent units (RFU) in a PolarStar Omega Spectrophotometer (BMG LabTech, UK). All assays were carried out in triplicate and are represented as means ± standard deviation. (DOCX) [file ppat.1010226.s001.docx]

**Supporting information**

**
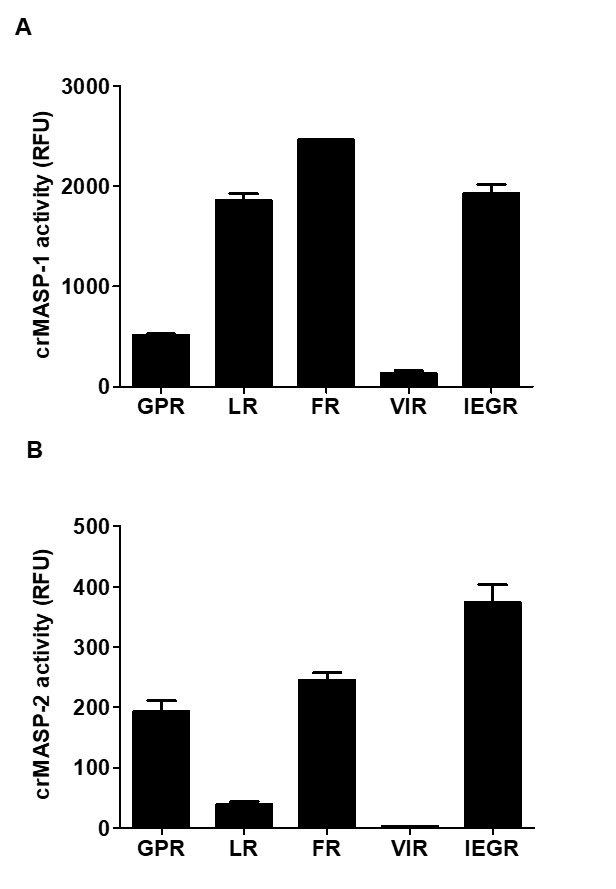
**

**S1 Fig. Screening of substrates to assess crMASP-1 and crMASP-2 enzymatic activity.** The activity of recombinant (A) crMASP-1 (0.1 µM) and (B) crMASP-2 (0.2 µM) was assayed with different fluorogenic substrates, namely Z-Gly-Pro-Arg-AMC (GPR, 40 µM; Bachem, UK), Z-Leu-Arg-AMC (LR, 40 µM; Bachem, UK), Z-Phe-Arg-AMC (FR, 40 µM; Bachem, UK), Z-Val-Ile-Arg-AMC (VIR, 40 µM; Bachem, UK), Z-Ile-Glu-Gly-Arg- AMC (IEGR, 40 µM; Bachem, UK). The proteolytic reactions were performed in TBS-Ca^+2^ (150 mM NaCl, 50 mM Tris, 20 mM CaCl2, 0.05% Tween-20 (*v/v*), pH 7.8) and measured continuously for up to 1 hr at 37ºC, as relative fluorescent units (RFU) in a PolarStar Omega Spectrophotometer (BMG LabTech, UK). All assays were carried out in triplicate and are represented as means ± standard deviation.
