# Supplementary material for: Fasciola hepatica is refractory to complement killing by preventing attachment of mannose binding lectin (MBL) and inhibiting MBL-associated serine proteases (MASPs) with serpins
Source: PLoS Pathog. 2022 Jan 10;18(1):e1010226. doi: 10.1371/journal.ppat.1010226 (PMC8782513; doi:10.1371/journal.ppat.1010226)
Supplement: S2 Fig — (A) NEJs 3 hr post-excystment in PBS. (B) NEJ 24 hr post-excystment which were kept incubated in RPMI medium, at 37°C with 5% CO2. (C) NEJ 24 hr post-excystment which incubated in 100% Normal Human serum (NHS), at 37°C with 5% CO2. Images were made using a light microscope (25x magnification). Scale bars, 10 mm. (DOCX) [file ppat.1010226.s002.docx]

**Supporting information**


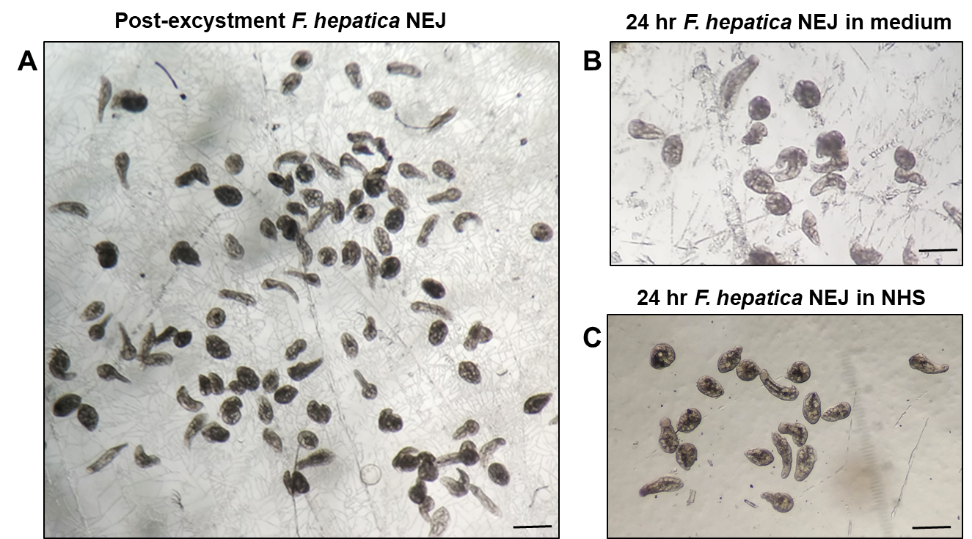


**S2 Fig. *F. hepatica* NEJ survive incubation in Normal Human Serum.** (A) NEJs 3 hr post-excystment in PBS. (B) NEJ 24 hr post-excystment which were kept incubated in RPMI medium, at 37°C with 5% CO_2_. (C) NEJ 24 hr post-excystment which incubated in 100% Normal Human serum (NHS), at 37°C with 5% CO_2_. Images were made using a light microscope (25x magnification). Scale bars, 10 mM.
