# Supplementary material for: Fasciola hepatica is refractory to complement killing by preventing attachment of mannose binding lectin (MBL) and inhibiting MBL-associated serine proteases (MASPs) with serpins
Source: PLoS Pathog. 2022 Jan 10;18(1):e1010226. doi: 10.1371/journal.ppat.1010226 (PMC8782513; doi:10.1371/journal.ppat.1010226)
Supplement: S3 Fig — Immunolocalization studies were carried out to assess complement deposition on the surface of whole mount NEJ cultured in RPMI medium, fixed and then incubated with NHS for 1 hr before being probed with anti-human MBL (1:250), anti-human C3b (1:250), anti-human C4b (1:250) or anti-human C5b-9 (MAC, 1:500). All samples were analysed by confocal laser microscopy represented by green fluorescence (FITC staining) and counter-stained with phalloidin-tetramethylrhodamine isothiocyanate (TRITC) for visualisation of the NEJ musculature (red fluorescence). The profile of immunolocalization is shown on two planes; on the surface of the NEJ (Outside) and internally (Inside). OS, oral sucker. VS, ventral sucker. Scale bars, 25 μM. (DOCX) [file ppat.1010226.s003.docx]

**Supporting information**


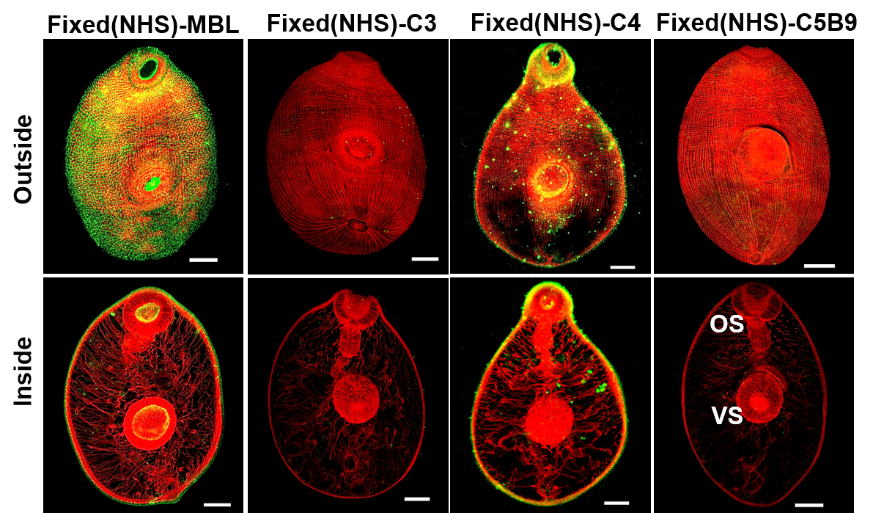


**S3 Fig. Complement deposition on the surface of dead *F. hepatica* NEJ following incubation in human serum.** Immunolocalization studies were carried out to assess complement deposition on the surface of whole mount NEJ cultured in RPMI medium, fixed and then incubated with NHS for 1 hr before being probed with anti-human MBL (1:250), anti-human C3b (1:250), anti-human C4b (1:250) or anti-human C5b-9 (MAC, 1:500). All samples were analysed by confocal laser microscopy represented by green fluorescence (FITC staining) and counter-stained with phalloidin-tetramethylrhodamine isothiocyanate (TRITC) for visualisation of the NEJ musculature (red fluorescence). The profile of immunolocalization is shown on two planes; on the surface of the NEJ (Outside) and internally (Inside). OS, oral sucker. VS, ventral sucker. Scale bars, 25 µM.
